# Supplementary material for: Functional Characterization of Muscarinic Receptors in Human Schwann Cells
Source: Int J Mol Sci. 2020 Sep 11;21(18):6666. doi: 10.3390/ijms21186666 (PMC7555815; doi:10.3390/ijms21186666)
Supplement: Supplementary file 1 [file ijms-21-06666-s001.pdf]

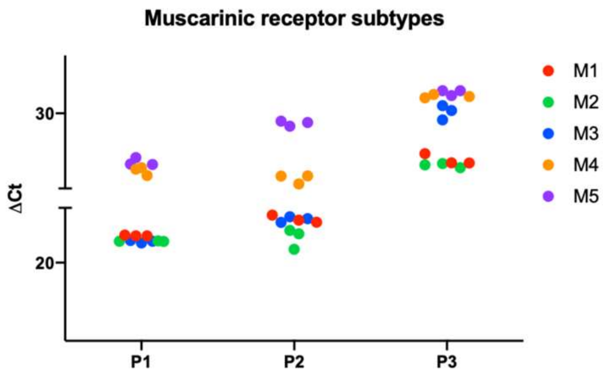

Figure S1. qRT-PCR analysis for muscarinic receptor subtypes. The graph reported the triplicate of the ΔCt for each muscarinic receptor subtypes in three different patients. The most express subtypes are M1 and M2 for each patient whereas M4 and M5 present a lowest expression; M3 appears more variable.

Table S1. Statistical analysis of MTS assay after APE and muscarine treatment at the final concentration of 100 μM.

Tukey's multiple comparisons test	*Summary*	*Adjusted P Value*
Ctrl 3DIV vs. APE 3DIV	****	<0.0001
Ctrl 3DIV vs. Muscarine 3DIV	***	0.0001
Ctrl 3DIV vs. Ctrl 5DIV	**	0.0065
Ctrl 3DIV vs. Ctrl 7DIV	****	<0.0001
APE 3DIV vs. APE 5DIV	ns	0.8367
APE 3DIV vs. APE 7DIV	****	<0.0001
Muscarine 3DIV vs. Muscarine 5DIV	****	<0.0001
Muscarine 3DIV vs. Muscarine 7DIV	****	<0.0001
Ctrl 5DIV vs. APE 5DIV	****	<0.0001
Ctrl 5DIV vs. Muscarine 5DIV	****	<0.0001
Ctrl 5DIV vs. Ctrl 7DIV	****	<0.0001
APE 5DIV vs. APE 7DIV	****	<0.0001
Muscarine 5DIV vs. Muscarine 7DIV	ns	>0.9999
Ctrl 7DIV vs. APE 7DIV	****	<0.0001
Ctrl 7DIV vs. Muscarine 7DIV	ns	0.985

One-way ANOVA statistical analysis followed by Tukey multiple comparisons post hoc-tests. The results showed a significant increase of cell growth between APE 3 and APE 7 DIV (**** $p < 0.0001$) or Muscarine 3DIV *vs* 5 and 7 DIV (**** $p < 0.0001$). Although this increase at different days of APE treatment, cell growth between untreated and treated cells at the same time point have shown a decrease of cell growth after M2 agonist exposure.
